# Breath-based lung cancer detection using an ML-driven low-cost sensor array

**DOI:** 10.1038/s41598-025-26416-z

**Published:** 2025-11-24

**Authors:** Dhruv Iyer, Kavin Gobinath, Krish Kowkuntla, Vitthalrao Vijaykumar Wanjari, Gokulakrishna Banumurthy

**Affiliations:** 1 Mountain View High School, Mountain View, 94040 US; 2 Irvington High School, Fremont, 94538 US; 3 Maharashtra Medical Council, Nagpur, India; 4 University of Alabama at Birmingham, Birmingham, 35294 US

**Keywords:** Cancer, Engineering, Medical research

## Abstract

Lung cancer is the leading cause of cancer-related mortality worldwide. Lately, electronic nose (e-nose) systems have emerged as a promising method for non-invasive lung cancer detection. These systems, however, have several limitations, including low accuracy rates and long detection times. To address these challenges, we conducted a pilot study involving the development of an affordable e-nose device that can detect more than 30 volatile organic compounds, using twelve metal oxide semiconductor sensors and one chemi-resistive alkane sensor. The device recorded data for 28 healthy controls and 18 lung cancer breath samples that were then analyzed using a multilayer perceptron neural network. The dataset was expanded through a novel use of data augmentation, where Gaussian noise was applied to generate synthetic samples while preserving the original data’s statistical properties. The model was evaluated by 5-fold cross-validation and achieved an accuracy of 96.26%, sensitivity of 92.88%, specificity of 97.75%, and an area under the curve of 0.9286. Our system outperforms existing e-nose detection methods by more than 5% and is capable of classifying in approximately 5 minutes. These findings highlight the potential of this breath analyzer system as a rapid and cost-effective tool for preliminary lung cancer screening.

## Introduction

Lung cancer is the deadliest form of cancer, accounting for 1 in 5 total cancer deaths^1^. Despite recent advances in detection, more than 75% of lung cancer patients are diagnosed after stage 3, when treatment options are limited and prognosis is poor^2^. However, early detection of lung cancer can substantially improve survival outcomes. For patients diagnosed at stage I, the 5-year survival rate can exceed 90%, whereas for those diagnosed at stage IV, the 5-year survival rate drops to 10% or lower^3^.

Although current lung cancer detection tools have high accuracy, they also have limitations. Low-dose CT (LDCT) scans are the most common, but they require expensive equipment and expose patients to radiation^4,5^. Additionally, a study conducted by the National Lung Cancer Screening Trial, which involved 53,454 participants, reported LDCT scans had a 96.4% false positive rate for early stages of lung cancer, highlighting another limitation of LDCT scans^6^. Tissue biopsies remain another standard method for accurate diagnosis, but due to their invasive nature, they carry associated risks^7–9^.

A promising diagnosis method for lung cancer utilizes gas sensors to analyze volatile organic compounds (VOCs) for breath-based detection^10–13^. Certain diseases, such as chronic obstructive pulmonary disease (COPD) and pulmonary fibrosis, can alter the composition of VOCs in human breath, allowing them to serve as biomarkers for those diseases^14–18^. This method is viable for lung cancer as it has a distinct VOC profile that does not overlap with other cancers or diseases^19–21^. This is the effect of the unique processes caused by lung cancer, such as altered protein expression, gene mutations, and the Warburg effect, which is an abnormal reliance of cancer cells on aerobic glycolysis^19,20,22^. These processes lead to the production of VOC byproducts such as toluene, benzene, and acetone, as well as the alkane VOC subgroup, which serve as analytical markers for detection^23–26^.

Currently, the gold standard for breath-based lung cancer detection is the gas chromatography-mass spectrometry (GC-MS)^27–30^. However, it is expensive, requires specialized lab equipment to operate, and has a lengthy processing time. Recently, many studies have used small-scale sensor arrays to detect and analyze various VOC compounds present in exhaled breath^31–33^. They show the promise of e-nose systems but are often limited by moderate classification accuracy, typically around 85%^34,35^. Additionally, some systems described can take up to 40 minutes to process one sample due to feature extraction, preventing them from being used for rapid detection purposes^36^. Fast results are especially valuable in primary care for screening during routine visits.

This pilot study aims to address these gaps by developing and validating an electronic breath analyzer system for highly accurate, rapid, and non-invasive lung cancer detection. In this pilot study, samples from 46 study participants were collected and analyzed using the constructed device. The dataset was then augmented to 79 samples. The resulting data were then used to train a multilayer perceptron neural network (MLP) to classify samples. Finally, 5-fold cross-validation was used to evaluate the model. Our system can provide results in under 5 minutes, underscoring its potential to serve as a preliminary screening tool to benefit both patients and clinical institutions.

## Methods

### Study design and population

This pilot study was designed to evaluate the effectiveness of a breath-based device for lung cancer detection. The study population consisted of 46 participants, including 28 healthy controls and 18 patients with a confirmed diagnosis of lung cancer. Data collection was conducted at the Rashtra Sant Tukdoji Regional Cancer Hospital (RSTRCH) in Nagpur, India. All participants provided one breath sample, which was collected in a 1L Tedlar gas sampling bag (CEL Scientific Corp., USA) and sent for analysis in the breath analyzer device^37^.

This study was approved by the RSTRCH Institutional Ethics Committee and performed in accordance with the Declaration of Helsinki. Written informed consent was obtained for each individual. were informed of the study’s purpose, procedures, and potential risks. The characteristics of the study population are presented in Table 1. The primary outcome measure was to evaluate the model’s accuracy in detecting lung cancer.

**Table 1:** Demographic and characteristics of participants.

	Lung Cancer (n=18)		Healthy Control (n=28)		p value
**Sex**	Male	9 (50%)	Male	11 (39.3%)	0.681
	Female	9 (50%)	Female	17 (60.7%)	
**Age (years)**		55.6 ± 11.5		41.2 ± 11.9	$<0.003$
**Stage**	I	6 (33.3%)			
	II	4 (22.2%)			
	III	3 (16.7%)			
	IV	5 (27.8%)			

### Selection of sensors

Based on prior research identifying lung cancer-indicative VOCs, we selected 12 gas sensors to capture a wide breath profile^23–26^. The device’s sensor array contains 12 metal oxide semiconductor (MOS) sensors and one chemiresistive alkane sensor. The alkane sensor was fabricated by following the procedures described by Tan et al. (2016)^38^. First, two copper electrodes were soldered onto a small printed circuit board (PCB) substrate, separated by a small gap. Then, 0.01g of tetracosane was deposited across the gap and melted at 100 degrees Celsius. Subsequently, 0.01g of carbon powder was melted on top of the tetracosane layer at 100 degrees Celsius. This process formed a stable, chemiresistive film that completes the circuit.

Table 2 shows the full list of sensors used in this study and their corresponding VOCs. The sensors primarily target different specific VOCs, with some compound cross-sensitivity. As a result, each sensor outputs a unique electrical resistance value when exposed to the same gas mixture^39^. This characteristic enables the sensor array to produce a distinct breathprint for control and disease groups, which can be used for analytical purposes^40,41^.

**Table 2:** Overview of gas sensors and their specifications.

No.	Model	Type	Range (ppm)	Detectable Gases	Manufacturer
1	TGS 2600	Metal Oxide Semiconductor	1–1000	Hydrogen, Ethanol, Formaldehyde, etc.	FIGARO
2	TGS 2602	Metal Oxide Semiconductor	0.05–10.05	Ammonia, Toluene, Hydrogen-Sulfide, etc.	FIGARO
3	TGS 2620	Metal Oxide Semiconductor	1–30	Ethanol, Toluene, Acetone, etc.	FIGARO
4	MQ-2	Metal Oxide Semiconductor	200-10,000	Benzene, Toluene, Xylene, etc.	Winsen
5	MQ-3	Metal Oxide Semiconductor	50-10,000	Ethanol, Acetone, Methanol, etc.	Winsen
6	MQ-4	Metal Oxide Semiconductor	200-10,000	Methane, Ethane, Propane, etc.	Winsen
7	MQ-5	Metal Oxide Semiconductor	200–2000	Benzene, Toluene, Propane, etc.	Winsen
8	MQ-6	Metal Oxide Semiconductor	200–1000	Butane, Propane, Hexane, etc.	Winsen
9	MQ-7	Metal Oxide Semiconductor	20–2000	Carbon-Monoxide, Benzene, Ethanol, etc.	Winsen
10	MQ-9	Metal Oxide Semiconductor	200–2000	Carbon-Monoxide, Methane, Benzene, etc.	Winsen
11	MC-135	Metal Oxide Semiconductor	10–500	Ammonia, Formaldehyde, Hydrogen, etc.	Winsen
12	Alkane Sensor	Chemiresistive Sensor	0–80	Alkane subgroup (Pentane, Benzene, etc.)	Made in Lab

### Design and fabrication of E-nose device

Our system is composed of two main components. The first is an airtight gas reaction chamber containing the sensors described in Table 2 and two fans positioned underneath the sensor plate to remove residual VOCs. Second, an electrical housing chamber holding the Arduino Mega 2560 R3 microcontroller (Arduino S.r.l., Monza, Italy), wiring, and battery that powers the device. The whole device costs approximately $215, with a more detailed cost breakdown shown in Table 3.

After a breath sample is transported inside the gas chamber, sensors react to the gases and output their resistance signals. Signals from the sensors are converted from analog to digital on the Arduino board and are sent to a computer through a universal serial bus (USB), where all time series voltage values are saved in comma-separated value (CSV) files.

**Table 3:** Cost breakdown of the E-nose device components.

Component	Approximate Cost (USD)	Description
Sensors (12 units)	$60	Main sensing elements (approx. $5 each)
Microcontroller & Electronics	$60	Arduino board, PCB, Dupont cables
Gas Chamber Housing	$40	Enclosure for sensors and electronics
Gas Sampling Pump	$30	To introduce breath samples into the chamber
Power Supply/Battery	$15	Rechargeable battery pack
Miscellaneous Parts	$10	Screws, tubing, adhesives, wiring
Total	$215	

**Fig. 1:**
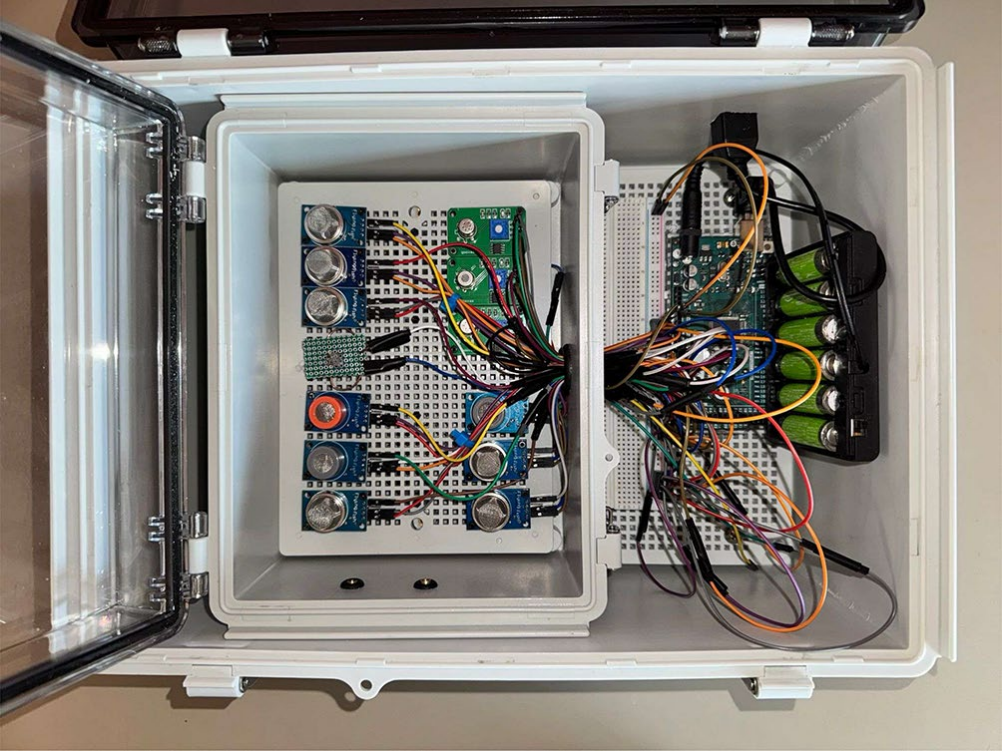
Photo of the e-nose system.

In order to collect and read sensor data from the Arduino, we developed a computer program written in Python to decode signals coming from the Arduino and save them into corresponding CSV files for each sample on a computer. An image of our device is shown in Fig. 1.

### Exhaled breath sampling process

Patient recruitment excluded those who had smoked or consumed alcohol within the last 24 hours. This was to ensure the patient’s VOC profile was not impacted by substances that could skew results^21,42,43^.

The sampling process starts with pumping ambient air into the gas chamber at a rate of 0.5-liter/minute, using a gas sampling pump. During this time, baseline calibration sensor readings were recorded for 30 seconds. Then, the patients breath sample is pumped into the airtight gas chamber, where it remains for 30 seconds. During the response phase, sensor readings were recorded at a sampling frequency of approximately 0.97 Hz, yielding 29 data points per sensor. This procedure ensured that sensor responses could be compared against a stable baseline for accurate discrimination.

**Fig. 2:**
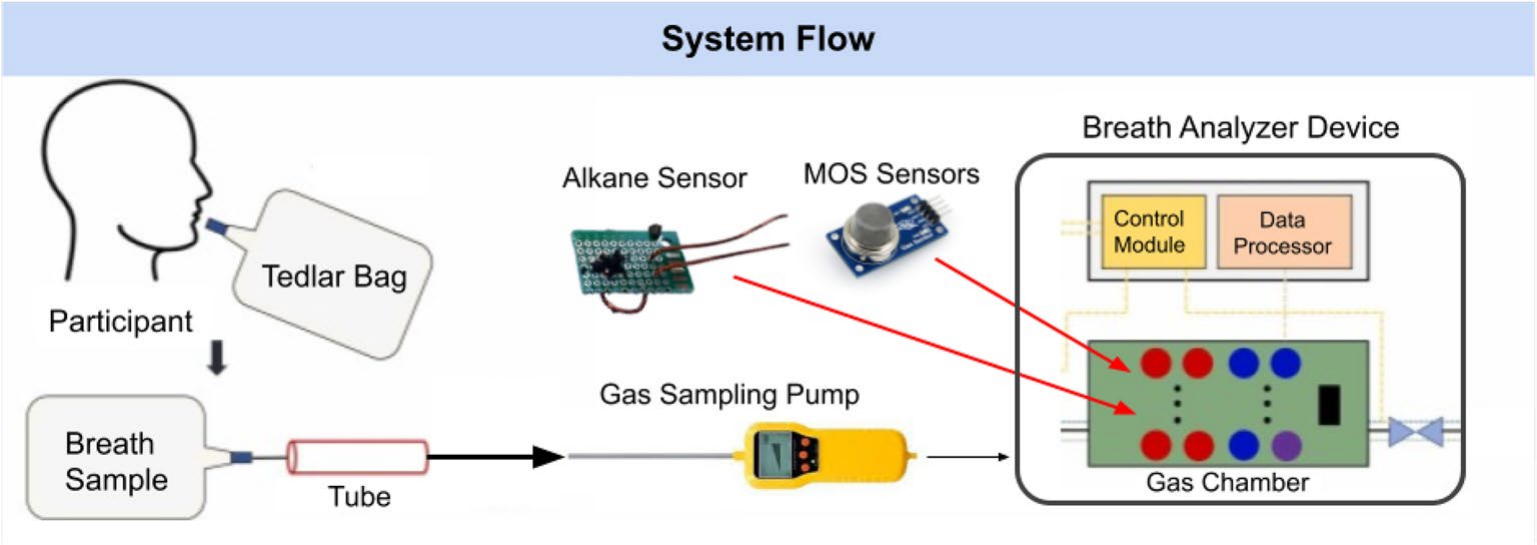
System flow of the proposed breath analyzer for lung cancer detection. Exhaled breath samples are collected into a Tedlar bag and introduced into the device with a gas sampling pump. The breath passes through an array of MOS and alkane sensors housed within a gas chamber, where the gas responses are measured.

After data collection, the gas chamber is cleared of the previous sample by opening its lid and turning on the internal fans to expel the air. The chamber is then resealed and filled with nitrogen gas. As an inert gas, nitrogen clears residual VOCs in the sensor cores through inelastic collisions without chemically interacting with the sensor core itself. This cleaning process is essential as it prevents previous breath samples from impacting subsequent readings and ensures a stable sensor baseline reading for consistency and accuracy. The cleaning cycle takes approximately 1 minute, and after this, the device is ready to process the next breath sample. A visual demonstration of this entire process is portrayed in Fig. 2.

### Data analysis and augmentation

Prior to model training, the resistance data obtained from the sensor array underwent several preprocessing steps: baseline correction, standardization, data augmentation, and dimensionality reduction. Baseline correction was performed by subtracting the mean resistance value recorded during the initial stabilization period from each sensor signal to account for environmental noise. Standardization was then applied to rescale all features to zero mean and unit variance, ensuring comparability across sensors.

Our dataset included 28 healthy and 18 lung cancer samples, which is small relative to the dimensionality of the sensor feature space (12 sensors $\times$ 29 time points $= 348$ features per sample). Small-*N*, high-*d* settings are susceptible to overfitting and unstable decision boundaries. To improve generalization of the model while retaining class structure, synthetic samples were generated by perturbing existing samples with isotropic Gaussian noise^44,45^,

$$\tilde{\textbf{x}}^{(\text {syn})} = \tilde{\textbf{x}}_i + \varvec{\varepsilon }, \qquad \varvec{\varepsilon } \sim \mathcal {N}(\textbf{0}, \sigma _a^2 I_d).$$

**Fig. 3:**
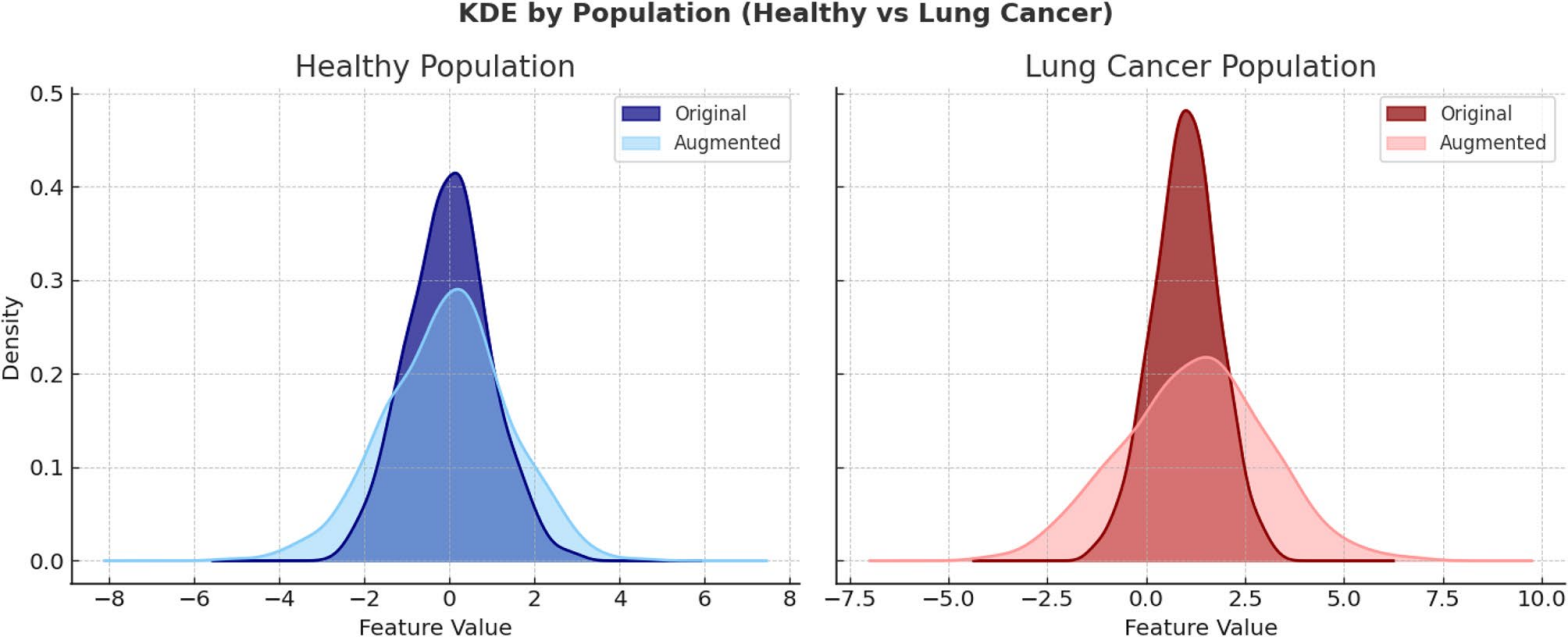
Distribution of a representative sensor feature after standardization for healthy (left) and lung cancer (right) populations. Dark colors denote original samples, while lighter shades represent augmented samples.

This preserves the class mean while inflating variance by $\sigma _a^2 I_d$, thereby modeling realistic variability without altering class centers. We generated $m_{\text {LC}} = 35$ synthetic lung cancer samples and $m_{\text {H}} = 25$ synthetic healthy samples for a final dataset of 53 lung cancer and 79 healthy samples. Only the training set was augmented to keep the test set strictly real. The noise amplitude was fixed at $\sigma _a = 0.6$ in standardized units. To verify that augmentation did not distort class structure, we performed two complementary checks:

1. **Univariate shape preservation:** For representative features, we compared kernel density estimates (KDEs) before and after augmentation. In standardized space, augmented curves broaden as expected (variance inflation) while retaining the location and modality of the original distributions (see Fig. 3).
2. **Two-sample equivalence testing:** For each feature and class, we applied the Mann–Whitney *U* test between the original and augmented samples. In all cases, the test yielded $p> 0.05$, indicating no significant difference between the two distributions and confirming that augmentation preserved the core statistical properties (consistent with Ultsch & Lötsch^46^).

## Results

**Fig. 4:**
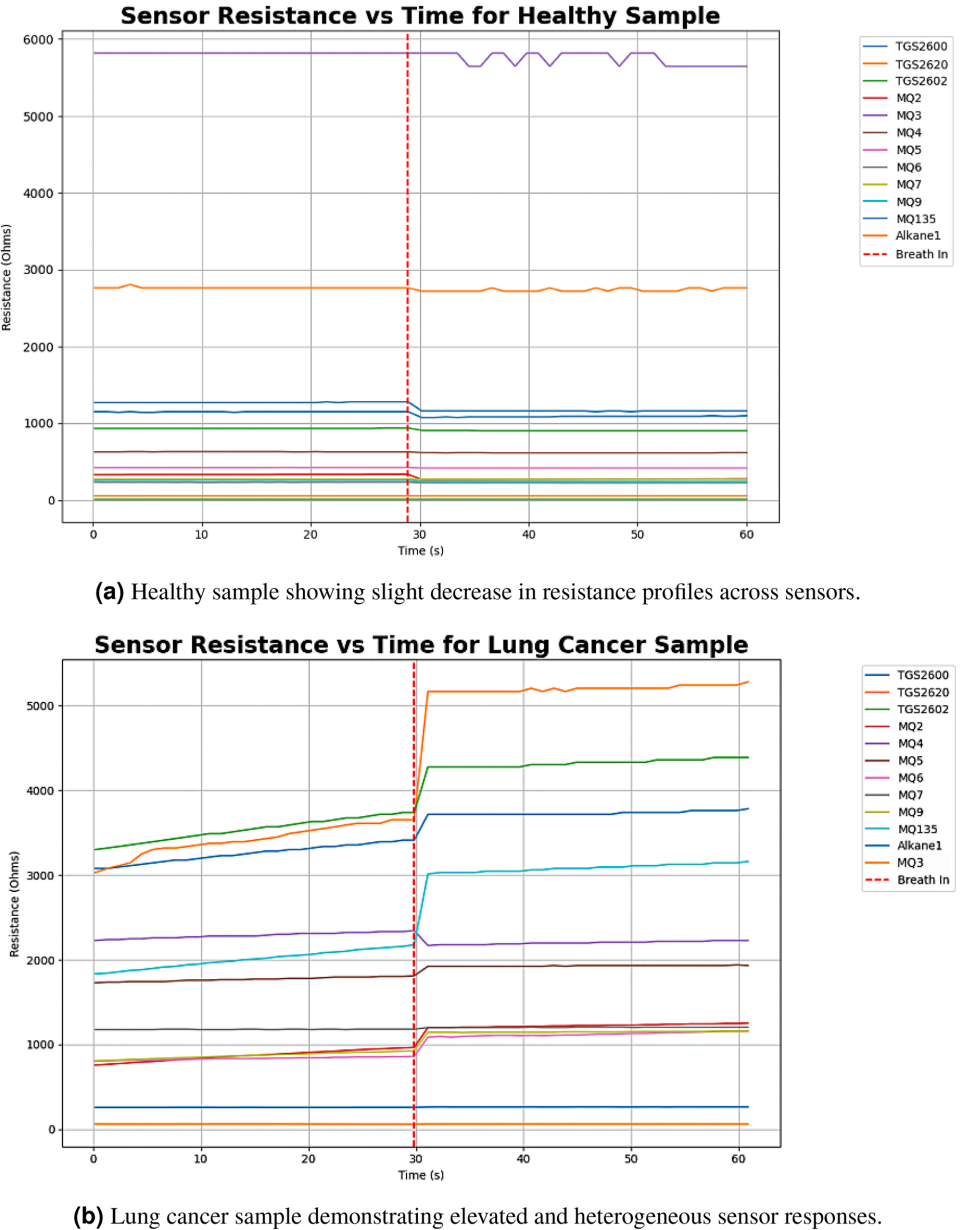
Representative sensor response patterns to exhaled breath.

Prior to deep learning-based classification, we analyzed sensor response data from healthy individuals and lung cancer patients. Fig. 4a shows the response of the gas sensor array to exhaled breath from a healthy individual, while Fig. 4b shows the responses to a breath sample from a lung cancer patient.

The two graphs show the responses of the 12 gas sensors, for which sensor-specific details are available in Table 2. Each sensor demonstrated distinct sensitivity profiles but comparable response times, with certain sensors–particularly TGS2620, TGS2602, and MQ135–exhibiting markedly higher responses to the exhaled breath of lung cancer patients. Variance in response and sensitivity was due to the different target VOCs of each gas sensor. This diversity in sensor reactions allowed for a broader breath profile to be captured, resulting in increased availability of information for the machine learning model to use for prediction.

**Fig. 5:**
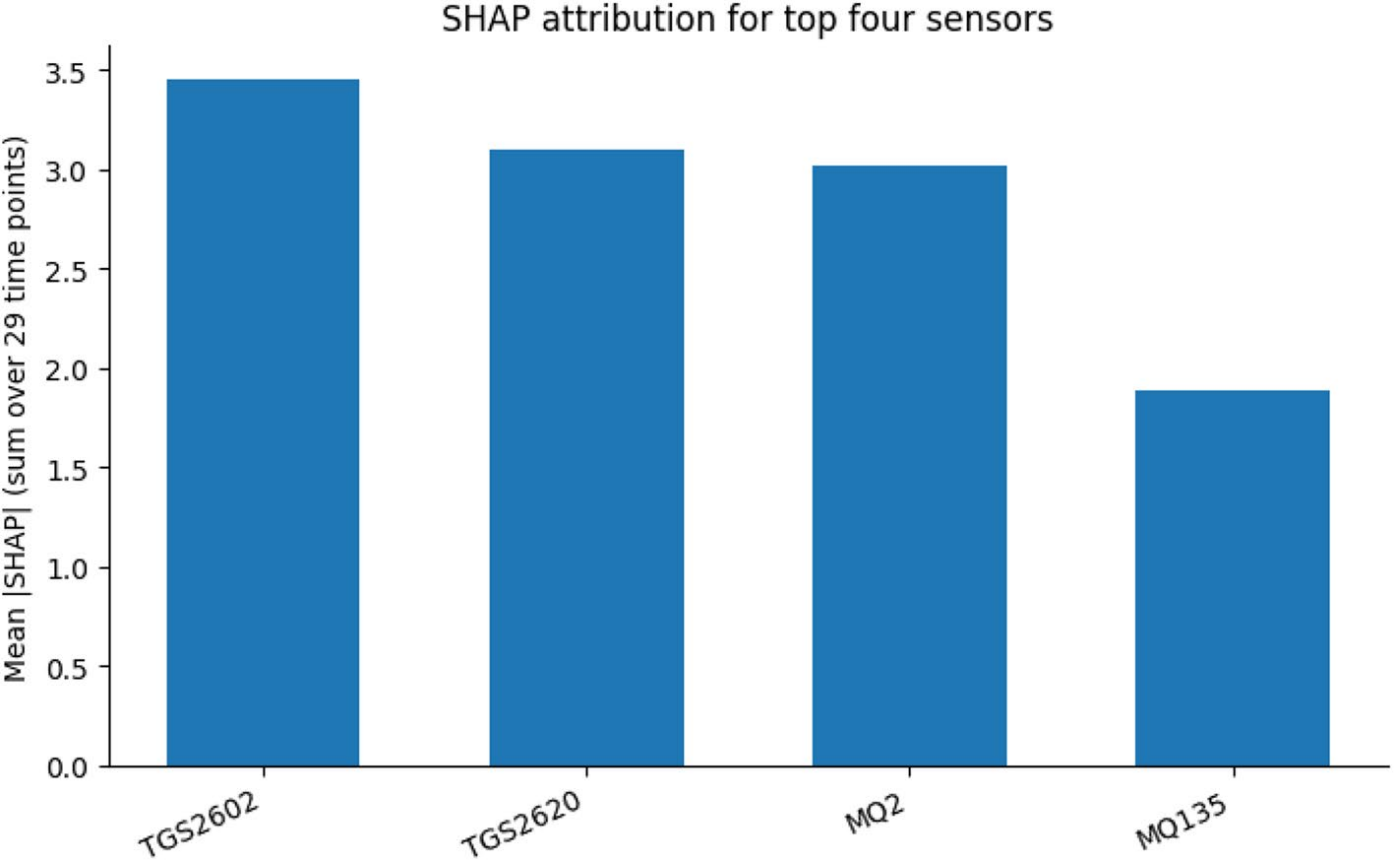
Cross-validated SHAP attribution for the top four sensors, averaged across folds and time points.

To further examine sensor performance, we performed a progressive backward ablation on the preprocessed 12-sensor matrix, using a stratified 5-fold cross-validation (CV) logistic-regression pipeline on 46 samples. Starting from all 12 sensors, at each step we removed the sensor whose removal yielded the highest CV receiver operating characteristic area under the curve (ROC-AUC). Performance peaked at $k = 4$ (mean CV AUC $= 0.9867 \pm 0.0267$), yielding the minimum-optimal subset ${\textrm{TGS2602}, \textrm{TGS2620}, \textrm{MQ2}, \textrm{MQ135}}$. For interpretability, we computed SHAP values on each CV fold, fitting the scaler and model only on the training data and evaluating SHAP on the held-out test fold to avoid information leakage. Per-sensor SHAP values were summed across all 29 time points and averaged over folds, yielding a robust measure of each sensor’s contribution (Fig. 5). The resulting SHAP ranking ($\textrm{TGS2602}> \textrm{TGS2620}> \textrm{MQ2}> \textrm{MQ135}$) aligned closely with the ablation-derived subset, reinforcing the discriminative importance of these four sensors. Sensors such as $\textrm{MQ9}$ and $\textrm{MQ6}$, which were removed early in ablation, consistently showed negligible SHAP contributions across folds, indicating functional redundancy despite differences in raw signal profiles.

**Fig. 6:**
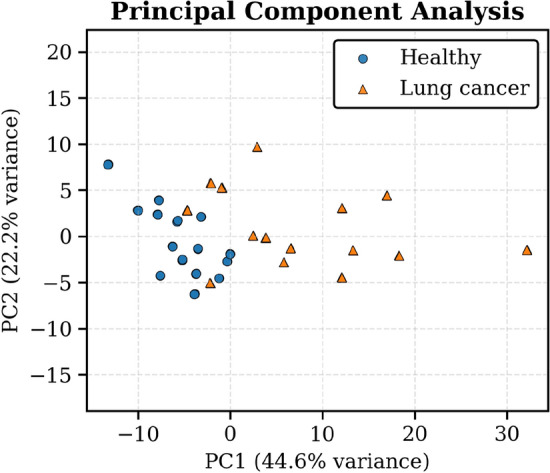
Two-dimensional mapping results of PCA. Blue circles represent samples from healthy individuals, and orange triangles represent samples from lung cancer patients.

To examine differences between the healthy and lung cancer sample populations, we applied principal component analysis (PCA) statistical modeling to the dataset. We projected the first two principal components (PC1 and PC2), which explain 66.8% of the total variance. Fig. 6 shows the resulting 2D scatter plot, where each point represents an individual breath sample, colored by class label. The plot shows distinct clustering as the lung cancer samples are generally separated from healthy samples along PC1. While there is some overlap between the two classes in the central region, most of the points are visually separated from each other. This suggests that the variance captured by the first two principal components reflects substantial statistical differences between classes.

A multi-layer perceptron (MLP) model was implemented to classify lung cancer and healthy breath samples using multimodal sensor array resistance data. The model was developed in TensorFlow and trained on a Google Colab environment with GPU acceleration.

Each breath sample was represented as a flattened 348-element vector, corresponding to 29 measurements from each of 12 chemiresistive sensors. The dataset consisted of 28 healthy and 18 lung cancer samples that were collected under controlled conditions. Synthetic sample generation through data augmentation expanded the dataset to 79 healthy and 53 lung cancer samples. Following this process, the data was split such that 70% of both healthy and lung cancer samples were used for training. The remaining 30% of samples were kept for testing. The training set was shuffled and used to train the MLP for 100 epochs with a batch size of 8.

The MLP architecture consisted of three fully connected hidden layers with 128, 64, and 32 neurons, respectively, each followed by ReLU activation, L2 regularization ($\lambda$ = 0.001), batch normalization, and dropout layers (dropout rate = 0.3) to mitigate overfitting. The final output layer contained a single neuron with sigmoid activation for binary classification. The Adam optimizer was then employed with a learning rate of $1 \times 10^{-4}$, and binary cross-entropy was used as the loss function.

To justify the choice of classifier, we benchmarked the MLP against other machine learning and deep learning models: Logistic Regression, Random Forest, XGBoost, and a convolutional neural network (CNN). These results can be seen in Table 4. 5-fold cross-validation was used for the evaluation of the models. The MLP delivered the best overall performance (accuracy $=0.963$; F1 $=0.956$) with strong sensitivity and specificity. Logistic Regression, Random Forest, and XGBoost clustered together and were noticeably lower than the MLP across metrics. Despite the clear separation suggested by principal component analysis, linear models (Logistic Regression) did not match the MLP, indicating that non-linear interactions across sensors and time points are informative. The CNN was more competitive with the MLP, but it offered no improvement in overall accuracy or F1 score and showed less stability. Additionally, its added architectural complexity increased the risk of overfitting without any clear benefit. For these reasons, the MLP was chosen as the primary classifier in this study.

**Table 4:** Classification metrics under five-fold cross-validation. Values are averages of all folds.

Model	Accuracy	Specificity	Sensitivity	F1-score
Logistic Regression	0.913	0.930	0.862	0.908
Random Forest	0.882	0.875	0.873	0.869
XGBoost	0.863	0.881	0.871	0.852
MLP	0.963	0.978	0.929	0.956
CNN	0.942	0.950	0.916	0.928

**Fig. 7:**
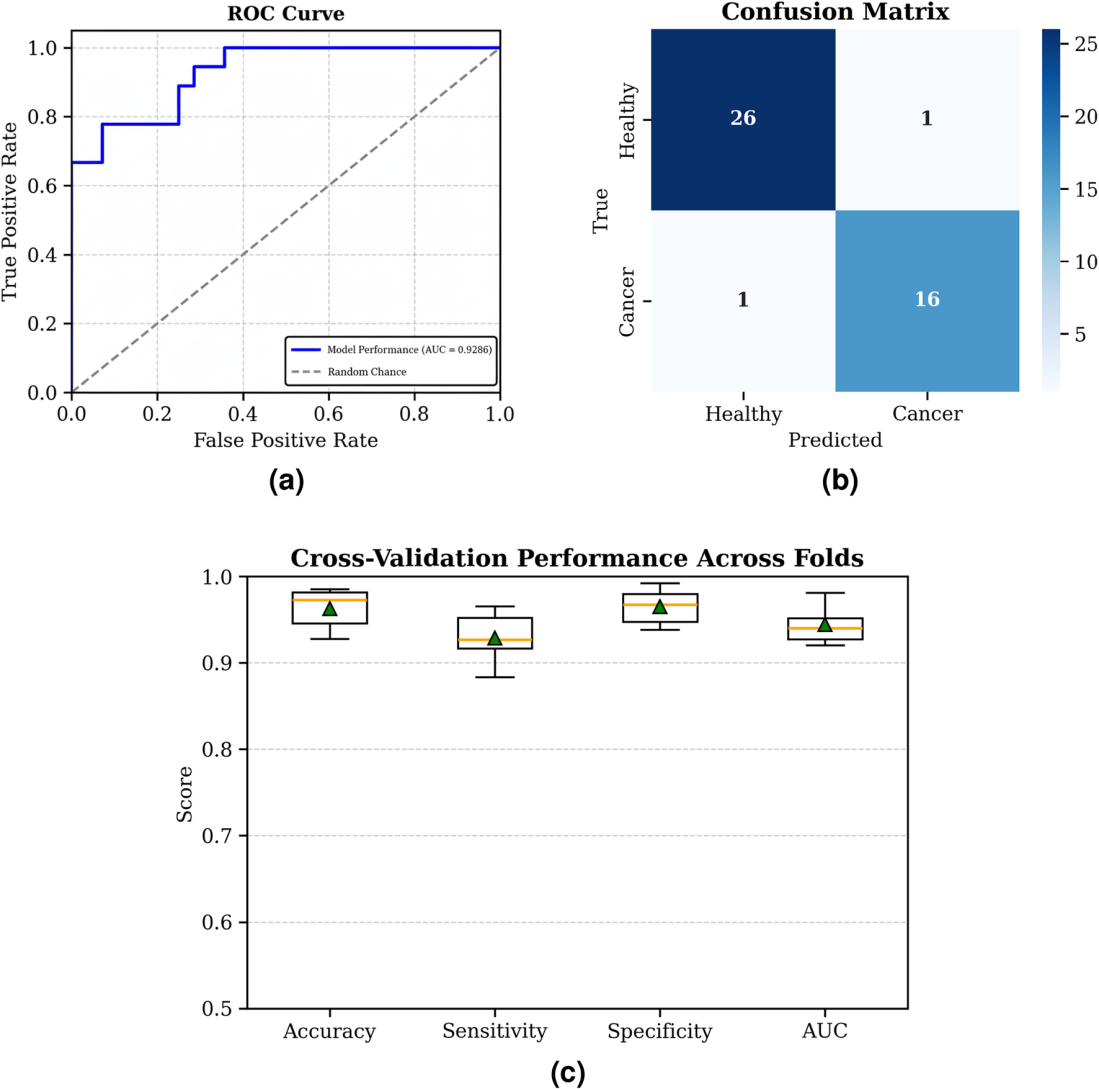
**(a)** Receiver operating characteristic (ROC) curve of the model, with an area under the curve (AUC) of 0.9286; **(b)** Confusion matrix of classification results showing correct predictions for healthy and cancer classes; **(c)** The performance metrics of the MLP model for classification of lung cancer patients and controls. All values are the averages of the results from 5-fold cross-validation.

Performance evaluation on the test set yielded an area under the ROC curve of 0.9286 (Fig. 7a). From this, the confusion matrix (Fig. 7b) demonstrated the model’s reliability, with only one false positive and one false negative on the test set. This corresponded to a sensitivity of 92.88% and a specificity of 97.75%, with an overall classification accuracy of 96.26%. The detailed cross-validation performance metrics are shown in Fig. 7c.

## Discussion

This pilot study demonstrates that an efficient breath analyzer combined with an MLP can accurately distinguish between lung cancer patients and healthy controls. The device achieved a mean area under the ROC curve (AUC) of 0.9286, an accuracy of 96.26%, a sensitivity of 92.88%, and a specificity of 97.75%. The high sensitivity and specificity signify the accuracy and precision in detecting lung cancer using this device. These performance metrics highlight the device’s potential as a rapid, non-invasive screening tool that could facilitate early detection, improve clinical outcomes by enabling timely intervention, and aid in primary care screening.

The device performance is competitive with existing multimodal sensor-based systems. Previous studies show accuracy rates between 85% to 92%. For example, Li et al. (2019) achieved 91.6% accuracy and Ozsandikcioglu et al. (2020) achieved a classification accuracy of 88.56%, both using a small-scale sensor array^34,47^. Our system’s accuracy of 96.26% outperforms these previous studies by roughly 5%.

A novel approach in our study is the use of data augmentation to overcome the challenge of limited clinical breath samples. This method enabled us to expand the training dataset while preserving its original structure, as confirmed by the Mann-Whitney U test (p> 0.05)^19,48^. Our model also did not overfit, indicating that our synthetic data exhibited ample variability. Additionally, our study incorporated a unique combination of small-scale MOS sensors, enabling the device to detect a broader range of VOCs. This contributed to the system’s higher prediction accuracy.

Our system is also original due to its fast prediction; the whole pipeline only requires 5 minutes from analyzing a patient’s sample to performing nitrogen cleaning. This marks a notable improvement over previous multimodal sensor studies with upwards of 40 minutes for prediction, as they absorb VOCs from exhaled breath instead of leaving the breath untreated^36^. Rapid detection using the system presented in this study offers particular value in primary care settings, where patients can be screened during routine visits. Unlike conventional blood or imaging tests, which require several days for processing and follow-ups, the breath analyzer system enables immediate identification of high-risk individuals during a quick in-office visit. By integrating such a device into routine check-ups, primary care physicians could expedite diagnostics, intervene earlier, and identify asymptomatic lung cancer cases, ultimately improving patient outcomes.

Accurate early-stage lung detection is another strength of the system. With the majority of the lung cancer group consisting of early-stage patients (stages I and II), the model achieved an average accuracy of 96.26% through 5-fold cross-validation. This demonstrates the consistency of our device and model across different training and testing sets, proving the system’s viability for early-stage lung cancer detection.

Lastly, our device is portable and does not depend on lab equipment, unlike other studies in this field. All these characteristics, combined with the device’s low cost of approximately $215, make our system more suitable for lung cancer preliminary screening in both clinical and non-clinical settings. It also has the potential to improve lung cancer detection in underserved regions where conventional diagnostic tools are limited or unaffordable.

While the device yielded promising results, there are a few limitations that should be acknowledged. The study population mainly consisted of South Asian individuals, and the study sample size was limited. Furthermore, the sensor array was largely homogenous, relying on MOS sensors.

Future work should aim to validate these findings in a larger, more diverse patient population. This presents an opportunity to further improve the system’s accuracy by researching the use of type-different sensors in our array, such as electrochemical or catalytic combustion sensors. Secondly, we implemented an MLP neural network; however, this can be expanded to evaluating other convolutional or recurrent neural networks for better performance. Additionally, a key challenge to widespread implementation will be gaining clinical adoption against established standards like LDCT or tumor biopsies, which will require further robust validation. However, we believe that the promising results of this pilot study provide an opportunity for further research to be conducted, thereby enhancing our understanding of the novel device’s utility.

## Conclusion

The findings display the potential of our small-scale sensor device for VOC breath analysis-based lung cancer detection. The device demonstrated high accuracy across all stages of lung cancer while remaining low-cost, portable, and capable of rapid analysis. These features suggest that the system could be deployed not only in hospitals and clinics but also in resource-limited and underserved settings, where access to affordable diagnostic tools is often limited. By enabling earlier detection, the device may help identify high-risk individuals prior to the development of symptoms and thereby support clinicians in developing more timely and effective treatment plans. In addition, the results provide evidence to inform large-scale screening initiatives, contribute to the standardization of breath-based diagnostic protocols, and advance a practical approach for further investigation. Collectively, these attributes position our system as a promising tool with the potential to significantly impact lung cancer prevention and early diagnosis in healthcare.

## Supplementary Information

Supplementary Information.

## Data Availability

The datasets generated during the current study are not publicly available due to the sensitive nature of patient medical information and restrictions imposed by patient consent. However, de-identified data may be made available from the corresponding author on reasonable request.

## Code availability

The Python scripts used for decoding and saving sensor readings to a CSV file database, data augmentation, and model training/evaluation are provided as Supplementary File 1.
